# A moving fluoroscope to capture tibiofemoral kinematics during complete cycles of free level and downhill walking as well as stair descent

**DOI:** 10.1371/journal.pone.0185952

**Published:** 2017-10-09

**Authors:** Renate List, Barbara Postolka, Pascal Schütz, Marco Hitz, Peter Schwilch, Hans Gerber, Stephen J. Ferguson, William R. Taylor

**Affiliations:** Institute for Biomechanics, ETH Zurich, Zurich, Switzerland; University of Memphis, UNITED STATES

## Abstract

Videofluoroscopy has been shown to provide essential information in the evaluation of the functionality of total knee arthroplasties. However, due to the limitation in the field of view, most systems can only assess knee kinematics during highly restricted movements. To avoid the limitations of a static image intensifier, a moving fluoroscope has been presented as a standalone system that allows tracking of the knee during multiple complete cycles of level- and downhill-walking, as well as stair descent, in combination with the synchronous assessment of ground reaction forces and whole body skin marker measurements. Here, we assess the ability of the system to keep the knee in the field of view of the image intensifier. By measuring ten total knee arthroplasty subjects, we demonstrate that it is possible to maintain the knee to within 1.8 ± 1.4 cm vertically and 4.0 ± 2.6 cm horizontally of the centre of the intensifier throughout full cycles of activities of daily living. Since control of the system is based on real-time feedback of a wire sensor, the system is not dependent on repeatable gait patterns, but is rather able to capture pathological motion patterns with low inter-trial repeatability.

## Introduction

It is well accepted that the accurate quantification of *in vivo* knee joint kinematics and kinetics is essential for a thorough understanding of knee functionality [1], particularly after total knee arthroplasty (TKA). Such knowledge allows the verification and improvement of new surgical procedures and rehabilitation techniques, the development of new concepts for knee implants and the advancement or validation of numerical models. In human movement analysis, skin marker based measurement is currently still the most employed tool. However, this method is critically affected by soft tissue artefact (STA), which reduces the ability to assess skeletal kinematics [2, 3].

In the past two decades, single plane videofluoroscopic analysis [4–9] as well as dual orthogonal fluoroscopy [10] have provided valuable information in the study of the three-dimensional motion of TKAs and normal knees. These technologies work by applying low-dosage radiographic imaging at sufficient frame rates to allow a dynamic assessment of the skeletal kinematics, and have the advantage that they are not affected by STA. Here, 3D registration of the 2D images is generally performed using CAD models of the implant components, or the segmented geometry from CT or MRI scans of the bones, together with a 2D/3D registration algorithm [5, 8, 11–14]. One drawback of this methodology, however, is the limited field of view of the generally stationary image intensifier, resulting in assessments of only highly restricted movements, as the knee must remain within the field of view of the image intensifier. Thus, past research has mainly focused on examining the kinematics of the knee during rising up from a chair (or sitting down), step-up/step-down and deep knee bend activities, often capturing only a portion of the whole motion [15, 16]. However, the negotiation of stairs is considered to be one of the primary daily activities that cause difficulties for subjects afflicted with knee disorders. Due to the larger joint moments [17], as well as higher variability and left-right asymmetries [18] compared to level walking, many TKA subjects develop compensatory movements to safely complete the task in a pain free manner. Given that downhill walking shows an increase in knee flexion, in the magnitude of the first peak of the vertical ground reaction force, in the braking force of the anterio-posterior component, as well as in knee joint moments [19, 20], it is no great surprise that walking down an inclined surface is also known to be challenging for TKA subjects.

Recent approaches suggest merging the information gained from subsequent videofluoroscopic and skin marker measurements [21–23]. Although this concept exploits the large measurement field provided by conventional skin marker gait analysis and the accuracy of videofluoroscopy, the limitations of STA and the restricted field of view of the static image intensifier still remain. The utilization of treadmills has also been proposed in order to confine the movement of the knee to the field of view of a video fluoroscope [24–26]. However, temporal [27–32], joint kinematic [27, 28, 30, 31, 33], as well as kinetic gait parameters [28, 31] are known to differ significantly between level and treadmill walking. Furthermore, individuals first need to be familiarized with treadmill walking [34], which can cause difficulties especially for elderly subjects or patients with a pathological gait pattern [31, 33].

A couple of research groups have been working on the development of mobile X-ray systems [6, 35–38], and recently Guan et el. [39] presented a mobile bi-plane X-ray system used for tracking of the knee joint during level walking. The system was capable of moving the X-ray units 3.6 m horizontally and 0.5m vertically at peak velocities of 5 m/s and 1.2 m/s respectively with peak accelerations of 20 m/s^2^ and 12 m/s^2^. Knee position was captured using a marker-camera sensor system, but the tracking of the system combined this data with a learned velocity profile. The resulting ability of the system to maintain the knee within the field of view was therefore dependent upon the repeatability of the motion task.

To overcome the limitations of a static image intensifier and allow tracking of the knee throughout complete cycles of level walking, Zihlmann et al. [9] presented a mobile fluoroscope that was capable of real-time tracking, thereby allowing natural variations in the gait cycle to be captured. One major advantage of the system is that it allowed the synchronous assessment of ground reaction forces. However, the system has not yet been assessed in its ability to accurately follow the knee throughout activities of daily living, and was only able to move horizontally. With the vision to allow the examination of more strenuous activities such as downhill walking and stair descent, the aim of this study was to further develop the system to additionally allow vertical tracking as well as to examine the accuracy with which the knee can be maintained within the field of view during different activities of daily living.

## Materials and methods

### The moving fluoroscope

**Fig 1:**
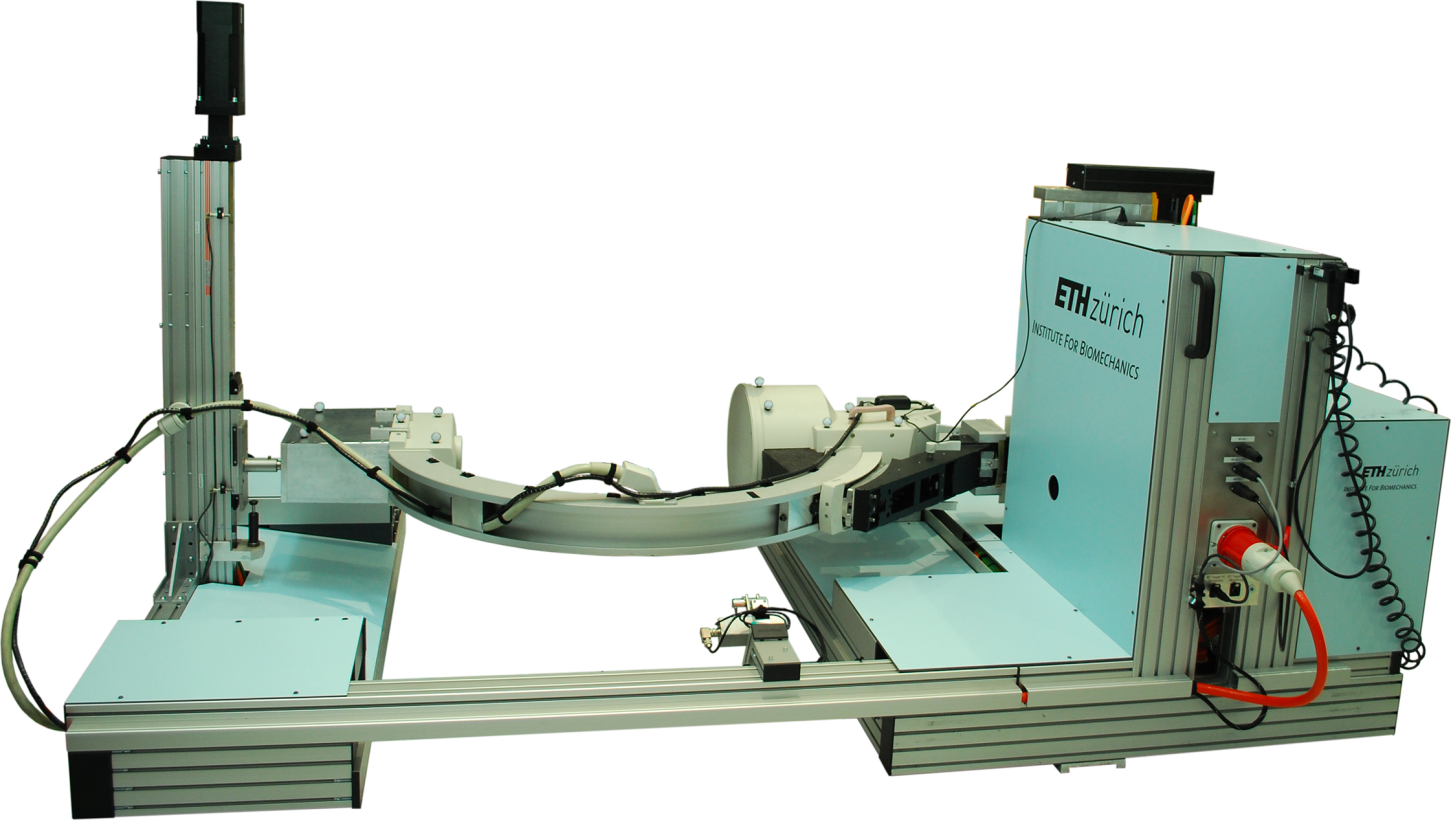
Moving fluoroscope developed at the Institute for Biomechanics. Consisting of a C-arm mounted on a moving trolley, allows real time tracking of the knee throughout complete cycles of level walking, stair descent and ramp descent activities. The figure shows the set up for measurements of the left knee. For measurements of the right knee, the C-arm is rotated by 180° around its out of plane axis and the front bar, including wire sensor is reconfigured to the other side of the trolley, such that the C-arm always leads away from the subject due to safety considerations.

**Fig 2:**
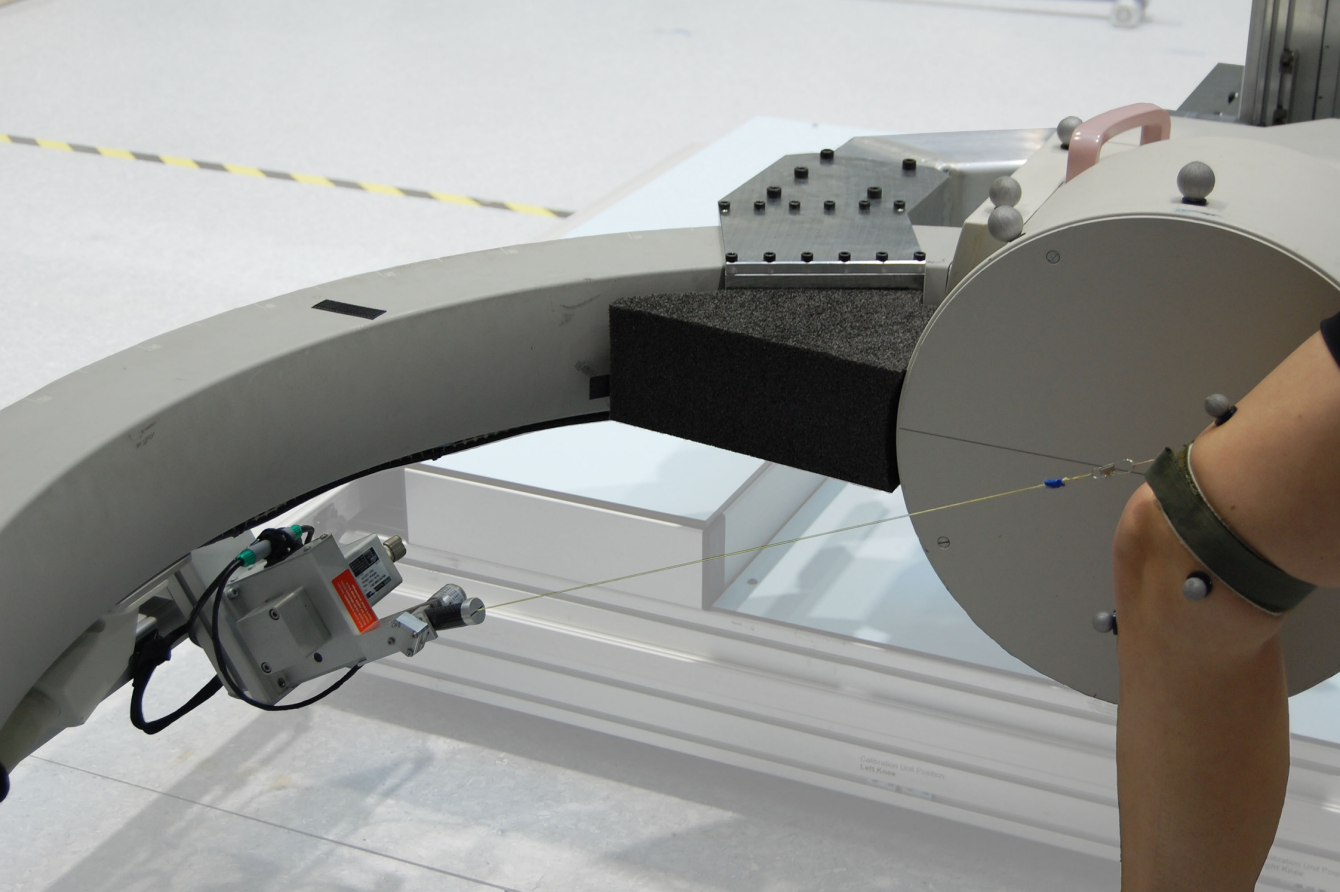
Wire sensor. Tracking of the trolley is achieved by positional feedback of the subject’s knee using a wire sensor and digital goniometer, thus allowing tracking of the knee joint both horizontally and vertically.

The moving fluoroscope has been developed to track the human knee joint over complete cycles of activities of daily living, and is composed of a modified BV Pulsera C-arm (Philips Medical Systems, Switzerland) mounted onto a moving trolley (Fig 1). The horizontal displacement of the trolley is achieved with six synchronous servo-motors (Mavilor BLS 113A, Infranor, Switzerland), which are controlled by six servo drives (XtrapulsCD1-a-400/45, Infranor, Switzerland), resulting in an ability to move the C-arm at up to 5 m/s with maximum accelerations of up to 9 m/s^2^. The vertical displacement of the C-arm is performed by two electrically geared and motorized (Movinor LH97, Infranor, Switzerland) linear actuators, one on the same side as the image intensifier (TKK30-325AL, Rexroth Bosch Group, Germany), and one on the side of the radiation source (LM4.2.0800BR020.1.04.0S, Line Tech AG, Glattbrugg, Switzerland). In this manner, the C-arm can achieve a maximum vertical velocity of 1.33 m/s and an acceleration of up to 4 m/s^2^. The relative location of the knee with respect to the trolley is measured using a wire sensor (WS17KT, ASM Sensor, Germany) in combination with a digital goniometer (05.2420.0020, Kuebler, Germany) that are attached to the leading bar of the trolley (Fig 2). The end of the wire is attached to an elastic velcro-strap that is secured around the thigh above the subject’s knee to be measured (Fig 2).

Based on the real-time excursion and inclination of the sensor wire, the motors were driven according to a feedback controller to keep the knee joint in the field of view (FoV) of the video fluoroscope both horizontally and vertically. Here, a PC running LabVIEW Real Time [LV 2014, National Instruments, Austin TX, USA] hosted a motion controller [PCI-7354, National Instruments, Austin TX, USA] and a DAQ-card [NI PCIe-6323, National Instruments, Austin TX, USA], which in combination with the software, transformed the knee sensor signal into vertical and horizontal coordinates for the motion controller. These calculations ran at a loop iteration frequency of 5 kHz. The vertical axis ran as a closed loop proportional integration controller in the displacement mode following the pre-determined set point. For the horizontal axis, the difference between the knee position and the pre-determined set point was used as a direct input signal for the motor drives, which were configured in velocity mode (a control range from 0 to 25 cm corresponded to 0 to 5 m/s).

Since the moving fluoroscope is mounted on a standalone trolley, horizontal walking distance is only limited by cable length and the existing lab configuration, thus allowing a range of about 10m. In the vertical direction, the moving fluoroscope allows tracking of the joint between 0.4m and 1.05 m above ground.

Finally, the fluoroscopy system was modified with the incorporation of a standalone charge-coupled device (CCD) camera (camera: IMPERX IPX-1M48-L, Imperx Inc., Boca Raton, USA; framegrabber: Matrox Solios eCL/XCL-B, Matrox Electronic Systems Ltd., Quebec, Canada) to allow a shutter time of 1ms and an image resolution of 1000x1000 pixels with a grayscale resolution of 12 bit. Imaging parameters of the X-ray system were: pulse frequency 25 Hz, pulse width 8ms, voltage around 60–70 kV and current around 10–12 mA.

### Coupled force, fluoroscope and reflective marker measurements

The video fluoroscopic image assessment was coupled with a motion capture system (VICON MX system, Oxford Metrics Group, UK) and force plates (Kistler, Switwerland). In order to time synchronize all three systems, a trigger signal was sent from the X-ray generator to an analog input of the motion capture system for each radiographic image.

The orientation and position of the fluoroscopy coordinate system was registered in the global laboratory coordinate system by using a grid of metal beads (also later used for image distortion correction), which was additionally equipped with six reflective markers affixed at predefined positions. The grid was rotated and displaced into multiple positions on the image intensifier, such that the grid’s beads were assessed by the fluoroscopic system while the locations of the reflective markers were simultaneously captured by the motion capture system. Since the positions of the reflective markers were identified in both the fluoroscopy and the laboratory coordinate systems, the transformation between the two coordinate systems was determined using a least-squares fit of point clouds [40]. Furthermore, to track the position of the moving fluoroscope during measurements, the C-arm was equipped with five reflective markers on the X-ray source and six on the image intensifier (Fig 1).

The setup of the force plates used for level walking consisted of five force plates fixed in a straight line, each with a dimension of 40 x 60 cm (2x Type 9281B, 2x Type 9285, 1x Type 9281C; Kistler, Winterthur, Switzerland). The force plates were decoupled from vibrations of the surrounding floor by installation on a separate concrete pillar constructed on the building foundation [9].

**Fig 3:**
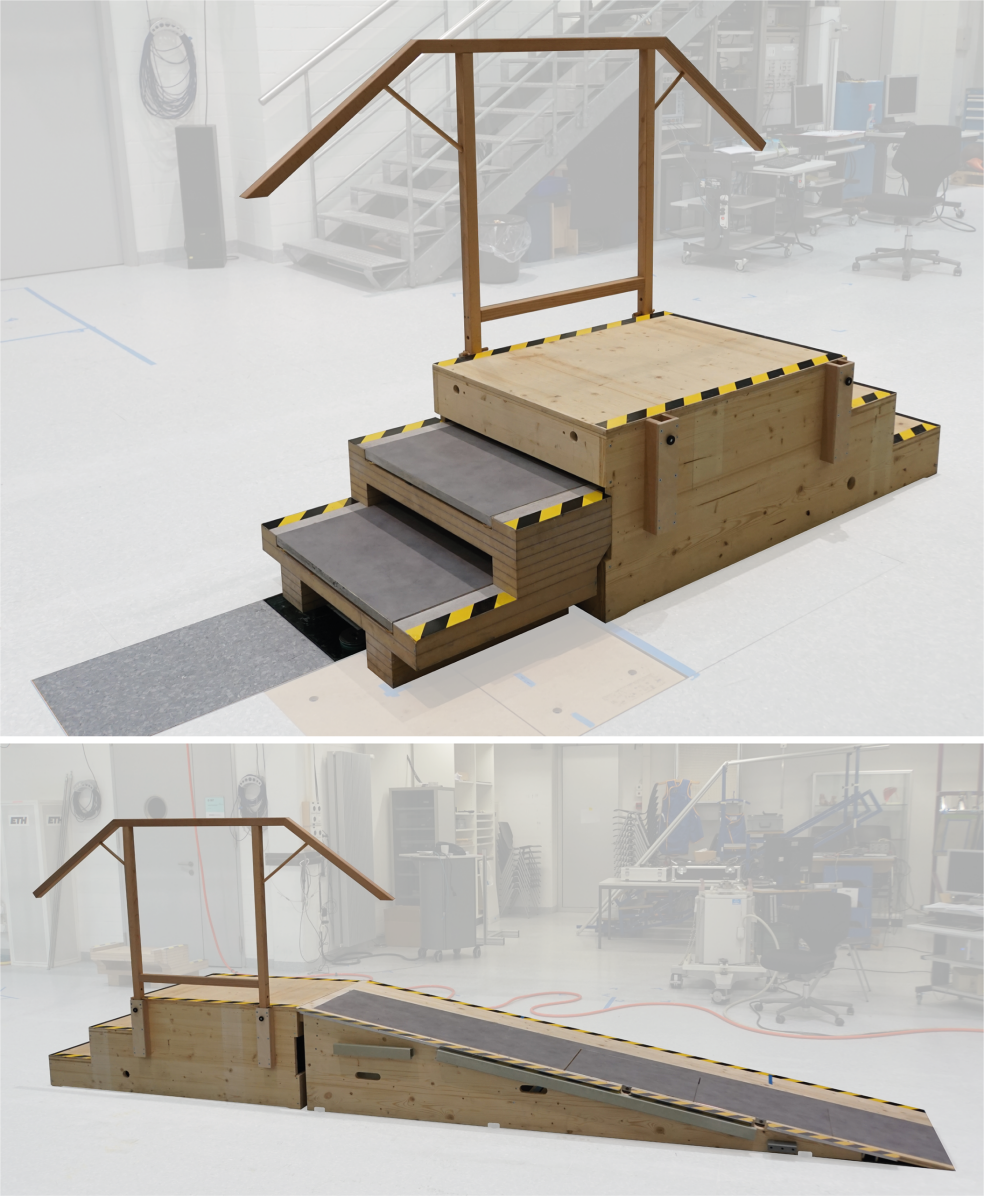
Instrumented staircase and ramp. Staircase and ramp (10° inclination) including two mobile force plates.

For stair descent, a three-step staircase equipped with two mobile force plates (Type 9286AA, Kistler, Winterthur, Switzerland), was fastened alongside the force plates fixed in the floor, thus also decoupled from any vibration in the surrounding floor. Each step of the staircase measured 280 x 180 x 800 mm in depth, height and width respectively (Fig 3).

For downhill walking, a ramp was constructed that included two mobile force plates (Type 9286AA, Kistler, Winterthur, Switzerland) that were also mounted onto the decoupled force plates in the ground (Fig 3).

### Feasibility study on 10 TKA subjects

The ability of the moving fluoroscope to track the knee within the image area was assessed in 10 TKA subjects (three female and seven male, six GMK PS fixed bearing TKA and four GMK Sphere TKA (Medacta International, Switzerland), at least one year postop, good outcome, no/very low pain with a Visual Analogue Scale score <2, average age of 69.6 ± 7.9 y and average BMI of 26.4 ± 2.9 kg/m^2^), who participated in an on-going study on TKA kinematics [41]. All subjects provided written, informed consent to participate in this study, which was approved by the cantonal ethical committee of Zurich (KEK-ZH-Nr. 2015–0140).

Prior to each experiment, each subject was given sufficient time to get accustomed to the moving fluoroscope without being exposed to radiation (two to four walking trials). For each motion task (level walking, stair descent, downhill walking) a minimum of 5 gait cycles were assessed. TKA kinematics by means of fluoroscopy, whole body kinematics based on skin markers, as well as ground reaction forces, were measured simultaneously. Data processing of the fluoroscopic images included image distortion correction by a local algorithm operating on a reference grid, assessment of the projection parameters of the video fluoroscopic system (focal distance, location of the principle point in the image plane) determined by a least-squares optimization using five images of a calibration tube, as well as 2D/3D registration based on the CAD models of the implant components [6, 11, 42]. In addition, the following parameters were computed to evaluate tracking capability of the moving fluoroscope:

#### Gait parameters

Gait velocity was calculated based on the midpoint between the two skin markers located on the posterior spina iliaca processes. Knee velocity and acceleration were tracked based on the knee markers on the lateral epicondyle.

#### Parameters reflecting motion of the moving fluoroscope

Maximal velocity and acceleration of the moving fluoroscope while tracking of the knee was evaluated in both the horizontal and vertical directions.

#### Tracking

The tracking was evaluated by the location of the knee joint centre, defined as the origin of the femoral implant coordinate system (mid point between medial and lateral epicondyles on femoral flexion axis), relative to the image intensifier.

## Results

The average gait velocity of the 10 subjects was 0.94 m/s for level walking (Table 1). In the horizontal direction for level and downhill walking, the maximal accelerations that were recorded by the moving fluoroscope were below the maximal knee accelerations, whereas the maximal velocities of the moving fluoroscope were larger than maximal knee velocities (Table 1). For stair descent, horizontal velocities and accelerations of the moving fluoroscope were comparable to the knee parameters. In the vertical direction, the maximal accelerations and velocities of the moving fluoroscope tended to be larger than those of the knee (Table 1).

**Table 1:** Gait Parameters and parameters of the motion of the moving fluoroscope.

	Gait Velocity		Knee				Moving Fluoroscope			
			Maximum Velocity		Maximum Acceleration		Maximum Velocity		Maximum Acceleration	
	[m/s]		[m/s]		[m/s^2^]		[m/s]		[m/s^2^]	
	horizontal	vertical	horizontal	vertical	horizontal	vertical	horizontal	vertical	horizontal	vertical
Level Walking	0.94 ± 0.11		1.87 ± 0.16		7.87 ± 0.72		2.06 ± 0.19		6.72 ± 0.41	
Stair Descent	0.51 ± 0.09	0.26 ± 0.03	1.20 ± 0.19	0.35 ± 0.30	4.61 ± 0.83	3.74 ± 0.97	1.18 ± 0.20	0.39 ± 0.24	4.62 ± 0.86	4.32 ± 1.14
Downhill Walking	0.78 ± 0.13	0.14 ± 0.03	1.59 ± 0.20	0.25 ± 0.19	6.93 ± 1.16	3.10 ± 0.60	1.73 ± 0.26	0.34 ± 0.10	6.54 ± 0.56	4.02 ± 0.80

Mean and SD over all 10 subjects.

The horizontal motion of the moving fluoroscope was stable in its trajectory, reflected by the absence of mediolateral motion (< 13 mm deviation in mediolateral direction from linear motion).

**Fig 4:**
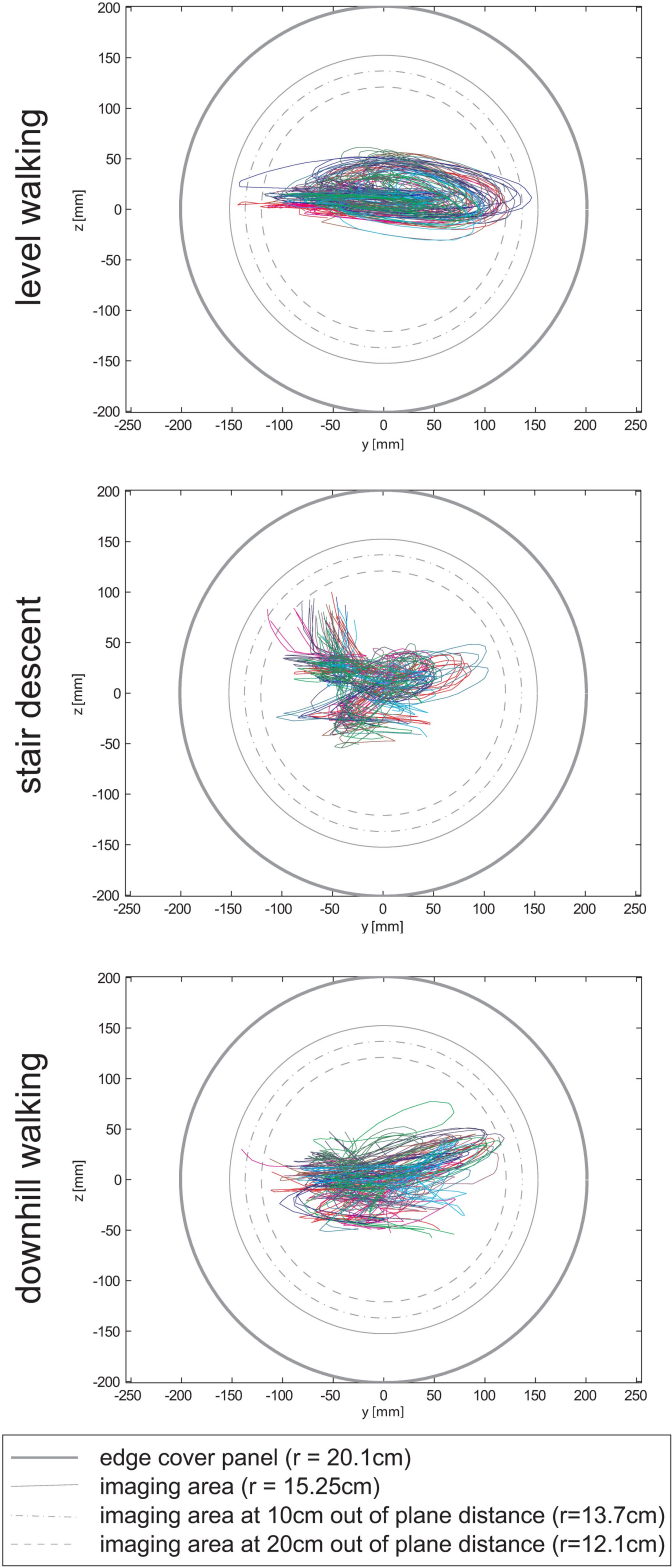
Tracking of the knee joint. Movement of the knee joint centre relative to the image intensifier during level walking, stair descent and downhill walking. All trials for all 10 subjects are shown (one colour for each subject).

The knee joint center for all ten subjects remained within the field of view of the image intensifier for all walking tasks and all trials, with an average distance between the knee joint centre and the centre of the image intensifier: vertical 1.8 ± 1.4 cm, horizontal 4.0 ± 2.6 cm (Fig 4, S1 Video).

## Discussion

The ability to reconstruct the skeletal kinematics of the human tibio-femoral as well as patello-femoral joints in 3D, without the influence of soft tissue artefact and throughout complete cycles of activities of daily living, provides critical new perspectives for understanding knee pathology and improving implant design. In this study, a moving fluoroscope has been developed and its ability to maintain the knee within the field of view of the image intensifier during level gait, stair descent and downhill walking has been demonstrated in 10 TKA subjects.

The construction of the moving fluoroscope as a C-arm mounted on a standalone trolley allows the usage within a variety of settings, since no construction or fixed installation on the floor is needed. In addition, the construction offers the ability to combine kinematic and ground reaction force measurements, and is only limited in walkway distance by cable length. The major disadvantage of this more mobile setup is the possibility of wheel slippage over the floor at higher gait accelerations, which can be overcome with optimal combinations of wheel and floor materials.

The presented moving fluoroscope is based on only a single C-arm, and therefore has both advantages and disadvantages compared to static systems or dual plane systems. Due to its dynamic nature, subjects are clearly aware of being accompanied by such a system during walking, both from a physical presence, as well as a noise perspective. As a result, gait velocities of the TKA subjects were lower than have been observed in healthy subjects [43, 44], but have been shown to become comparable to TKA subjects [45]. A previous study on subjects that were actively tracked by a robotic imaging system found only small and clinically non-relevant adjustments in gait patterns [37]. However, although gait velocity as well as ground reaction force patterns of the present study were comparable to TKA subjects of earlier skin marker studies, a future study should investigate if there is any influence of the presented moving fluoroscope, especially of the sound of the fluoroscope on the gait pattern of subjects walking with the moving fluoroscope. Another aspect of a single plane fluoroscope compared to a dual plane counterpart is the lower level of 2D/3D registration accuracy achievable, especially in the out-of-plane direction [46]. While this presents a considerable disadvantage, especially in the registration of natural bones, the approach also has clear advantages. A mobile single plane system provides a larger effective field of view than a static dual plane system. Dual plane systems physically constrain the measurement volume in two directions, thus confining the natural motion of the subject. Radiation exposure is also a critical, yet often neglected consideration; dual plane systems expose the subject to substantially higher levels of radiation. In this study, we have demonstrated the ability of the system to maintain the knee within the field of view (both horizontally and vertically) during a variety of activities of daily living–and this has been made possible by the wire sensor that provides a direct contact with the body of the subject. While the influence of the wire on gait is expected to be very small (it only applies an external force of about 4N), a clear advantage is that the time delay is small enough to allow real-time control. In other systems [39] tracking includes a learned velocity profile and since, especially for pathological gait, gait repeatability can be reduced, this can lead to deficiencies in being able to keep the knee in the field of view.

Due to the weight of the system, the presented real-time tracking is certainly not perfect. For a few frames, in which the knee joint centre reaches a location close to the edge of the image intensifier, part of the implant was out of the field of view. Although the information was sufficient to perform a 2D/3D registration, it is clear that if less information is available, reduced accuracy must be expected. Furthermore, any motion of the TKA relative to the image intensifier leads to the possibility of motion blur and therefore reduced accuracy in the 2D/3D registration. In the presented setup, however, the rapid shutter time of 1ms decreases motion blur, but a small amount of motion blur is still visible (see S1 Video), resulting in a lower Image Quality Measure [47] for some images during gait trials in comparison to static standing trials. As an example, the Image Quality Measure based on De and Masilamani [47] showed a range of 0.0006–0.0015 for the images of the gait trial presented in S1 Video in comparison to the Image Quality Measure of 0.0011–0.0012 in the images of a standing trial of the same subject.

The accuracy of the resulting tibiofemoral kinematics after 2D/3D registration is clearly influenced by the image quality and the registration algorithm itself, but is also strongly dependent on the object geometry, thus type and size of the implant and joint investigated. Previous validation of the aforementioned 2D/3D registration algorithm resulted in rotational and translational RMS errors of 0.2° / 0.4 mm for in-plane and 1.3° / 2.1 mm for out-of-plane for the Mobility^TM^ total ankle arthroplasty [42], and 0.15° / 0.3 mm for in-plane and 0.25° / 1mm for out-of-plane for the balanSys® TKA [6]. Other studies using single plane systems have reported slightly higher errors of between 0.3° to 1.4° and about 0.5 mm for in-plane rotations and translations and between 1.5° and 2.3° and between 4.0 and 6.6 mm for out-of-plane rotations and translations [5, 8, 46]. A similar accuracy can be expected for the two implant designs and various implant sizes included in the present study. For natural tibiofemoral kinematics, the errors are expected to be larger due to lower contrast and less prominent geometric features in comparison to TKAs.

## Conclusions

A moving fluoroscope has been presented that is a standalone system, and which allows the kinematic assessment of the tibio-femoral and patella-femoral joints by means of single plane videofluoroscopy during level gait, stair descent and downhill walking in combination with synchronous reflective marker and ground reaction force measurements. Its ability to keep the knee within the field of view of the image intensifier has been demonstrated in 10 TKA subjects. Since the fluoroscope is moved based on real-time control, its usage is not dependent on gait repeatability and will allow the evaluation of healthy as well as pathological gait patterns without the requirement for the joint to follow pre-determined paths.

## Supporting information

S1 Video Videofluoroscopic video of the knee, as well as time synchronized whole body skin marker and ground reaction force data during level walking. Furthermore, the trigger signal that was sent from the X-ray generator to an analog input of the motion capture system for each radiographic image, is displayed.
(AVI)
